# Direct 3D-Printed Orthodontic Retainers. A Systematic Review

**DOI:** 10.3390/children10040676

**Published:** 2023-04-03

**Authors:** Efthimia Tsoukala, Ioannis Lyros, Apostolos I. Tsolakis, Michael P. Maroulakos, Ioannis A. Tsolakis

**Affiliations:** 1 Department of Orthodontics, National and Kapodistrian University of Athens, School of Dentistry, 11527 Athens, Greece; 2 Department of Orthodontics, School of Dentistry, Case Western Reserve University, Cleveland, OH 44106, USA; 3 Department of Orthodontics, School of Dentistry, Aristotle University of Thessaloniki, 54124 Thessaloniki, Greece

**Keywords:** additive manufacturing, 3D direct-printed retainers, 3D orthodontic retainers, 3D-printed retainers

## Abstract

Three-dimensional (3D) printing technology has shed light on many fields in medicine and dentistry, including orthodontics. Direct 3D-printed prosthetics, implants or surgical devices are well-documented. The fabrication of orthodontic retainers using CAD technology and additive manufacturing is an emerging trend but the available data are scarce. The research approach of the present review included keywords in Medline, Scopus, Cochrane Library and Google Scholar up to December 2022. The searching process concluded with five studies eligible for our project. Three of them investigated directly 3D-printed clear retainers in vitro. The other two studies investigated directly 3D-printed fixed retainers. Among them, one study was in vitro and the second was a prospective clinical trial. Directly 3D-printed retainers can be evolved over time as a good alternative to all the conventional materials for retention. Devices that are 3D-printed are more time and cost efficient, offer more comfortable procedures for both practitioners and patients and the materials used in additive manufacturing can solve aesthetic problems, periodontal issues or problems with the interference of these materials with magnetic resonance imaging (MRI). More well-designed prospective clinical trials are necessary for more evaluable results.

## 1. Introduction

The term orthodontic retention refers to the means used by the clinician to prevent tooth relapse from the optimal aesthetic and functional condition after the end of the orthodontic treatment. In fact, retention is part of the orthodontic treatment [1,2,3]. The teeth have a tendency to return to their initial situation. Age changes in soft or skeletal tissues, occlusal limitations, pressure from cheeks, lips or tongue or tension from the periodontal fibers can lead to some degree of relapse [1,3,4,5,6]. For this reason, a proper retention protocol should be applied to every patient. In particular, the retention phase is more imperative for young patients, who have remaining growth potential even after the end of the active orthodontic treatment.

The most frequently used devices for retention can be divided in two categories. The first category includes removable retainers. These can be made from acrylic plate and stainless-steel wire work such as the broadly known Hawley-type appliance (HR) or Begg or Barrer-type, which is more common among children, or from transparent thermoplastic material, the vacuum-formed retainers (VFR) [7]. The second category are the fixed retainers bonded at the lingual surfaces of the upper or lower anterior teeth [1,3,7,8]. These are made up of plain or multistranded stainless-steel wires, nickelium/titanium wires or fiberglass in different thicknesses and designs. Although aesthetic and compatible with allergies or MRI artifacts, fiberglass splints have deteriorated mechanical properties in contrast to their metal analogs [8,9,10,11,12,13,14,15,16].

However, as dentistry is undergoing a technological revolution and is moving to a digital era, orthodontic retention should also be considered in this way [17,18].

In 1980, Hideo Kodama of Nagoya Municipal Industrial Research Institute in Japan invented 3D printing. Three years later, Charles (Chuck) Hull invented stereolithography (SLA), and in 1987 he also introduced the first 3D printer, the SLA-1^®^, to the market through his own company ‘‘3D Systems, Inc. (Rock Hill, SC, USA)’’ [17,18]. Nowadays, the first step into this digital workflow is the acquisition of data from intraoral scanners or cone beam computed tomography (CBCT). These data are then turned to standard tessellation language (STL) files and uploaded in a software program for 3D processing and designing of the device of choice. The next step is the uploading of the digital file on the 3D-printer digital platform. The most common types of printers used in dentistry are stereolithography (SLA), which utilize beams of UV light for the curing of photopolymers, direct light processing (DLP), which uses a light projector that projects slides of the 3D object to cure the resin, and material jetting (MJ), a new promising printing method. Printed materials vary among ceramic, metal or thermoplastic resin. The last step is post-manufacturing modification to ensure the proper construction of the product [17,19].

Three dimensional (3D)-printed dental appliances directly from a digital file, such as 3D-printed surgical splints, implant guides and indirect bonding transfer trays or biomedical and pedodontics appliances have been reported [17,20,21,22,23]. CAD technology and 3D printing have also introduced new tools and materials for the direct 3D-printing fabrication of either of the two types of retainers [24]. Intraoral scanners produce 3D digital models that so far have many diagnostic and treatment planning applications [21,22,25,26]. The 3D-printed models can also serve as plaster analogues for the thermoforming procedure of plastic retainers; however, directly 3D-printed clear or fixed retainers are not well established in clinical practice.

Some clinical reports have been made [27,28,29]. The first attempt for direct fabrication of a 3D-printed clear retainer was mentioned in 2014 by Nasef et al. from a computed tomography (CBCT) scan with satisfying results [30]. Moreover, a milling method from PEEK material was used by Zachrisson in 2018 to digitally fabricate a new type of fixed retainer [24]. Nowadays, retainers made of PEEK can be fabricated either with milling or printing technology [24,29]. PEEK material (poly-ether-ether-ketone) seems promising due to its biocompatibility, which could address issues such as allergies and MRI artifacts or nickel–titanium (NiTi) archwires covered in PEEK could also address esthetic concerns. However, more research is necessary to investigate other aspects such as the mechanical properties or the cytotoxicity of this polymer [31].

The interest towards direct 3D-printed fixed or clear retainers can be explained, as the new technologies in dentistry allow in-house fabrication capabilities. In-house scanners, 3D printers at low cost and free software that designs different kinds of orthodontic appliances are now available. This technological evolution has given the capability to the clinician of delivering to the patient their personalized retainer on the same day as the removal of the orthodontic appliances [25,32]. Marsh et al. have reported the use of the in-house virtual bracket removal (VBR) technique, a software that digitally removes the braces from the intraoral scan to produce a 3D-printed model for fabrication of the clear thermoformed retainers that facilitates same-day delivery [32]. It is important to mention that children often lose their retainers or they destroy them. As a result, they have to revisit the orthodontist to create new retainers. Three-dimensional technology allows the dentist to have an electronic storage of the final casts and to easily print the retainers for the patients. This way patients do not need multiple appointments to receive their retainers.

Therefore, the step forward in this technological breakthrough is to virtually design the retainers, fixed or clear, on the 3D digital models and directly print the retainers from the 3D printer, in this way eliminating the extra step of printing the 3D dental models and fabricating the retainers on them secondarily.

In this systematic review, the aim was to gather all the available data in the literature concerning directly 3D-printed retainers. However, a meta-analysis was not feasible because the clinical and experimental available data were not comparable due to the heterogeneous study design.

## 2. Materials and Methods

**Protocol and registration**

This study is designed and conducted according to the Preferred Reporting Items for Systematic Reviews and Meta-Analysis (PRISMA) Statement [33]. The protocol for this systematic review was registered on the Open Science Forum Database (Protocol: 10.17605/OSF.IO/6W2KU).

**Search Strategy**

Four Databases (Medline (via Pubmed), Scopus, Cochrane Library and Google Scholar) were thoroughly searched by two authors (ET and IT) for studies up to December 2022. There was a language limitation for English studies only. The key words used in the search strategy were «3D-printed retainers», «three-dimensional orthodontic retainers», «3D direct-printed retainers» and «additive manufacturing» (Table 1).

**Eligibility Criteria**

The eligibility criteria were applied in accordance with the PICO research approach, as follows:

- Population/Patients: post-treatment human dental arches or their digital models.
- Intervention: 3D-printed orthodontic removable or fixed retainers.
- Comparison: conventional removable or fixed Retainers or no comparison.
- Outcome: successful fitting and efficiency (retention, oral health and longevity) of the device.

**Study Selection and Data Extraction.**

Inclusion criteria were prospective or retrospective clinical trials, observational studies and in vitro studies. Exclusion criteria were reviews, authors’ opinions, thesis articles, case reports and case series. Duplicate studies were eliminated after all the retrieved studies were incorporated into the Mendeley reference management system (Elsevier, 2021). Two authors chose the studies separately and twice (ET, IL). Any discrepancies were clarified by conversation with a third author (IT). The authors, institutions or research conclusions of the studies were not kept a secret from the two authors. Following the reading of abstracts and the elimination of non-eligible papers, possibly pertinent studies were identified by title. Following this step, a manual search of the eligible studies’ references was conducted to uncover any undiscovered new papers. After thoroughly reading the articles, a decision was taken in accordance with the prerequisites.

**Figure 1:**
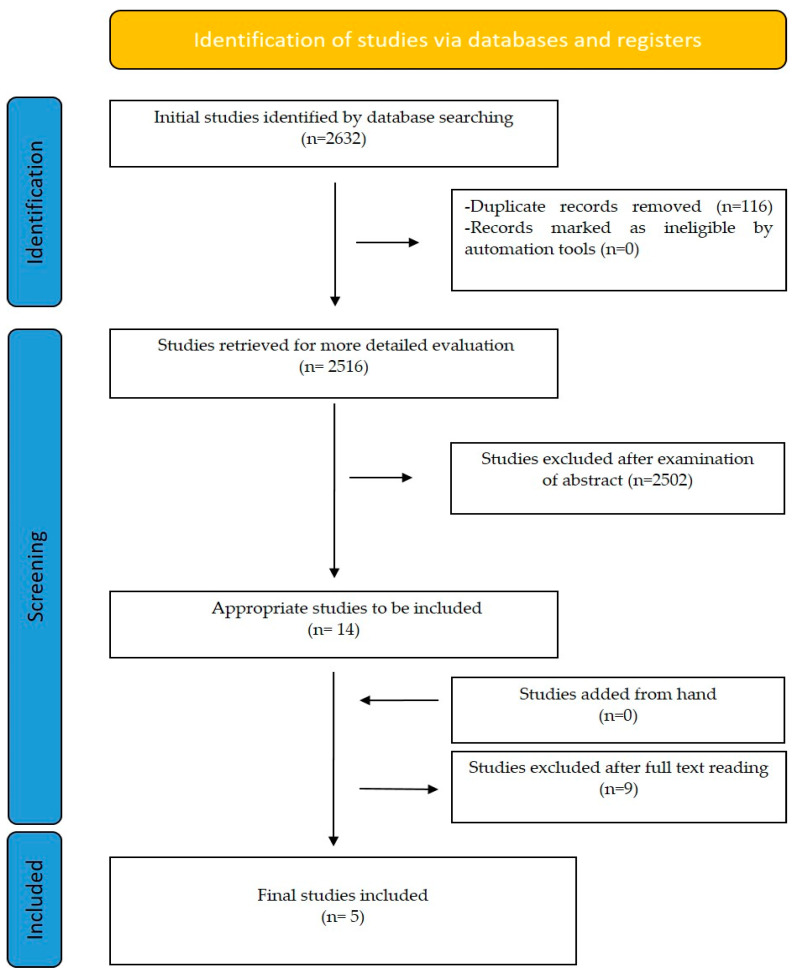
Prisma flow chart selection of records.

Initially, 2632 articles were retrieved. After duplicate removal and title and abstract screening by the two authors, 14 articles remained for full text evaluation. Finally, 5 articles were included in the analysis (Figure 1).

## 3. Results

Out of the five studies included, three evaluated the characteristics of direct 3D-printed clear retainers and all three were in vitro studies [21,22,34]. The other two evaluated the characteristics of direct 3D-printed fixed retainers. The study of Firlej et al. [24] was an in vitro study and the one performed by Shah et al. [35] was an in vivo randomized prospective clinical trial.

***Risk of bias assessment***

To assess the risk of bias, the Quin tool for the in vitro studies [36] and the RoB 2.0 tool [37] for the prospective randomized clinical trial according to the Cochrane guidelines, were applied. The risk of bias assessment was independently performed by two review authors (ET and IL). Any disagreement was resolved by a third author (IAT).

Table 2 displays the analytical results of the quality assessment of the risk of bias with the Quin tool and Table 3 shows the results of the quality assessment with the RoB 2.0 tool [37]. All of the studies were characterized by a high risk of bias. The in vitro studies exhibited a high risk of bias mainly considering the detailed explanation of sample size calculation, detailed explanation of sampling technique, details of the comparison group, operator details, randomization, blinding and presentation of the results. The in vivo clinical trial was also characterized by a high risk of bias, considering mainly the randomization process, the missing data and the selection of the reported result.

***Study Characteristics***

3D-Printed Clear Retainers

The 3D-printed clear retainers were assessed regarding fitting or accuracy, trueness and precision. Before the review of the studies, it is good to clarify the terms accuracy, trueness and precision [41].

Accuracy: The closeness of agreement between a quantity value obtained by measurement and the true value of the measurement.

Precision: The closeness of agreement between the independent test results obtained under stipulated conditions.

Trueness: The closeness of agreement between the average value obtained from a large series of test results and an accepted reference value.

In other words, high accuracy is a combination of high precision and high trueness.

Cole et al. [21] compared the accuracies of nine 3D-printed retainers with equal number of both traditional vacuum-formed retainers (TVF) and commercially obtainable vacuum-formed retainers (CVF) fabricated by Invisalign (Align Technology, San Jose, CA, USA) (29 in total). To determine the fit of the retainers, reference points were set at the 3D models. With the help of engineering software, superimposing of the digital images of the printed retainers and their original models was used to calculate the differences in these marked points. The results showed that TVF retainers had the most accurate fit, with the least difference for almost all the reference points measured, followed by CVF retainers. The largest variations from the reference models were measured for the 3D-printed retainer group. However, this last group showed the least difference between the reference points and 3D models at the incisal edges and cusp tips. The «weak points» of the 3D-printed retainers were the smooth surfaces of all teeth. However, all of the measurements for all three groups were below 0.50 mm, which is an «accuracy threshold» of a digital articulation, according to the study.

Naeem et al. [22] compared the accuracies of fifteen 3D-printed clear retainers fabricated by four different commonly used 3D printers: SLA (stereolithography), digital light processing (DLP), continuous light processing (cDLP) and polyjet photopolymer (PPP) printers. Six reference points and intercanine (ICW) and intermolar width (IMW) were compared after digital superimposing of the 3D digital models and the digital data of the printed retainers. The «accuracy threshold» according to this study was set at 0.25 mm. According to the results, all four kinds of 3D-printed retainers were below this tolerance level of accuracy. More precisely, the PPP printed retainer had the least deviations from the reference models at the region of the incisors, the DLP group at the region of the canines and the cDLP and SLA groups at the region of the molars. Regarding the ICW and IMW, the PPP printers resulted in the most accurate replication of width, followed by the SLA printers. A greater width discrepancy was observed for the DLP and cDLP printer groups. The lower inter-arch distortion of the PPP printers may be attributed to the horizontal orientation of printing in this group, as opposed to the other three groups of printers that printed with an angulation, or the smallest printing height of the PPP printers. Estimated from all aspects, his study concluded that PPP and SLA printers were the most accurate and DLP and cDLP were the most precise for printing retainers.

Williams et al. [34] studied the accuracies of sixty different 3D-printed clear retainers, printed at various angulations of 15°, 30°, 45°, 60° and 90°. All were printed from an SLA printer and used clear resin for the fabrication (Clear, Formlabs Inc., Somerville, MA, USA). Again, like Naeem et al., eight specific landmarks were set as reference points for digital superimposing and a 0.25 mm accuracy threshold was set as a measure of clinically acceptable accuracy. Their results showed that there is not a unique optimal print angulation. Overall, all print angulations were found to fabricate retainers within an accuracy threshold (from 0.074 to 0.225 mm) at the cusp tips and the incisal edges. However, smooth facial surfaces exhibited differences up to 0.480 mm and were not appraised as clinically agreeable. The most time-effective printing (1 h and 30 min) was observed when fabricating one retainer at 15° angulation. Printing at 60° and 90° took up to 2 h and 15 min. The most cost-effective printing was that at 45° angulation with 5.20 mL of resin required per retainer, whereas printing at 30° resulted in the most amount of resin required overall.

3D-Printed Fixed Retainers

Firlej et al. [24] examined the mechanical properties of 3D-printed fixed retainers. The retainers were digitally designed and printed in predetermined dimensions of 3 mm width, 30 mm length and in three different thicknesses of 0.8 mm, 1 mm and 1.2 mm. The resin used was Nextdent MFH C&B N1, a commonly used resin for printing restorations and bridges. The printer was a Phrozen MINI4k printer. The subject of the study was to examine whether the thickness of the retainers affected their strength under loaded conditions. Aging in artificial saliva at 37±°C was performed too. Flexural strength, elastic properties, deflection and creep were evaluated. It was found that the thickness of the material plays a crucial role in the mechanical properties of the retainer. All of the properties examined (flexural modulus, deflection, strength and creep) were superior for the 1.2 mm retainer sample. Surprisingly, the 1.0 mm sample had the inferior properties. This indicates that the mechanical properties and the width of the 3D printing resin are not directly proportional. After the aging process, it was also assumed that under simulated loading and humidity oral environment, the properties of resin-based materials are deteriorated and only the 1.2 mm sample retains its properties for a longer period of time. These first results concerning the mechanical characteristics of 3D-printed fixed retainers, revealed that they can satisfyingly replace metal retainers. It is also easy, cheap and fast to reproduce new ones in case of the breakage of the previous ones. However, this was an in vitro study and additional long-term clinical trials should be conducted for further evaluation.

Shah et al. [35] compared usual and customized 3D-printed fixed lingual retainers in terms of periodontal health, amount of relapse and failure. His study was a prospective randomized clinical trial. He randomly divided a group of 34 patients into two. The first group received a conventional multistranded braided stainless-steel fixed orthodontic lingual retainer; however, there was no reference to the company name of the product. The second group received a customized 3D-printed lingual retainer, with no reference to the 3D printer or resin used. The retainers were bonded at time T0, and the patients of each group were recalled after 3 months (time T1) and 6 months (time T2) of bonding for the evaluation of plaque index (PI), gingival index (GI), bleeding on probing (BoP), microbial load and P. Gingivalis estimation. The estimation of the amount of relapse was recorded via Little’s irregularity index (LII) using a digital Vernier caliper. The estimation of incidence of failure was recorded for wire breakage or bond failure at time T1 and T2 or whenever they occurred. The results showed that there were significant differences between the two types of retainers in plaque index, gingival index and bleeding on probing. The 3D-printed retainer was superior to the conventional one relating to periodontal health. However, neither of the two cultures collected from the conventional retainer group or the 3D-printed group were detected to contain P. gingivalis, indicating no chronic periodontal disease. A greater irregularity index (LII) was found for the group of conventional fixed retainers at time interval of 6 months. No wire breakage was reported for any of the two groups during the study. Bond failures occurred for both types of retainers, and between the two, more failures were reported for the conventional fixed retainer group. Therefore, for all aspects studied, the 3D-printed fixed retainers proved to be better.

## 4. Discussion

Removable retainers, either VFRs or HRs, provide better oral hygiene opportunities to the patient [42,43,44]; however, the literature so far contends that the difference between fixed and removable retainers in influencing the long-term development of periodontitis is insignificant [45,46,47,48,49,50,51,52,53,54]. In contrast with fixed retainers, removable retainers need lifelong compliance and cooperation from the patient [2,12,46,47,55].

Comparing the removable appliances, HRs provide better settling of the occlusion compared with VFRs, and they also seem to be more durable in terms of breakage [7,48,56,57]. Bowen Li et al. concluded in their systematic review that patients using HRs showed healthier periodontal condition than those using VFRs [58]. In contrast to this, VFRs are more aesthetic, do not interfere with speech and are therefore more acceptable to the patient [55,59,60]. Additionally, VFRs are more cost effective and easier to manufacture [7,48,56,61]. Moreover, they seem to provide better retention of anterior teeth irregularities [38,39,62]. However, both VFRs and HRs seem to maintain sufficiently orthodontically corrected intercanine and intermolar widths [57,63,64,65].

Other adjunctive procedures have also been reported that enhance the stability of the orthodontic result [66]. Among them, circumferential supracrestal fiberotomy and interproximal reduction are the more prevalent for the moment [5]. Moreover, pharmacological agents have been mentioned in the literature, but they are still in the experimental stage. Raloxifene has been proven to decrease post-orthodontic treatment relapse in vitro [67]. Results with the injection of bisphosphonates in vitro also suggested enhanced post-treatment stability [68]. Similar results have been suggested by Li et al. [69] with intermittent parathyroid hormone administration, by Duliamy et al. [70] with local injection with strodium, by Al-Fakhry et al. [71] with injectable platelet rich fibrin (i-PRF) administration and by Zhao et al. [40] with local OPG (osteoprotegerin) gene transfer to periodontal tissues. Ozturk et al. [72] investigated the effect of photobiomodulation and high frequency mechanical vibration three-dimensionally and molecularly and found that both can support post-treatment retention. The supportive effects of vibration for orthodontic retention were also highlighted in the study of Yadav et al. [73]. Though promising, all these procedures need further clinical investigation in order to be implemented.

It seems that orthodontists have plenty of options so far for the retention phase of the treatment. Although efforts have been made through the years for the standardization of the retention protocol, this is impracticable. Every patient needs tailormade approaches according to their age, pre-treatment characteristics, periodontal and dental situation, compromises during treatment, compliance, etc., [12,74,75,76]. Knaup et al. suggested the application of both removable and fixed appliances to enhance anterior teeth alignment stability over time [77].

Now that CAD-CAM and 3D-printing technology have found their application in orthodontics, it is just a matter of time for commercially available direct 3D-printed aligners and retainers to be released.

However, this review of the available literature proved that directly 3D-printed retainers are still at their infancy. Either fixed or removable clear, the retainers from 3D-printing technology so far are mainly resin based. So, a possible reason for this retardation is that there is no affirmed biocompatible resin that is commercially obtainable for this purpose [63,73].

EnvisionTEC (EnvisionTEC, Inc.; Dearborn, MI, USA) and Formlabs have announced the release of novel resins for the fabrication of clear aligners and occlusal splints, respectively, with appropriate biocompatibility and physical properties [21,34]. PEEK polymer is a material that has been used in some clinical case reports for fixed retention [29,78]. It is already known for its application in dentistry for implants or prosthetic restorations, but it also seems to be a promising material for orthodontic purposes due to its superior physical, mechanical and aesthetic properties, biocompatibility, low plaque affinity and flexural modulus close to enamel and dentin. However, more clinical research is necessary [31,79]. A case report also referred to an experimental resin (Genial Printing Resin, GC) from GC Orthodontics (GC America Inc., Alsip, IL, USA) for 3D printing of fixed retention [28].

The studies so far are mostly in vitro experiments or case reports of «laboratory-stage» products. Further well-designed clinical trials examining the properties and the behavior of 3D-printed retainers in long time periods are necessary.

One aspect to be examined in long-term clinical studies is the behavior of resin over time. It is known that resin-based materials become more fragile over time. The humidity of the oral environment, the chemical and temperature imbalances from food or beverage consumption, the stress loading during mastication or other factors also affect the properties and the behavior of the resin [24].

Leaching from 3D-printed resin is also one more aspect that should be examined. There are a number of biocompatible resins available for use in many fields such as in medicine and in dentistry [80]. However, the cytotoxic potential of 3D resin products is not yet well researched as the information available is scarce on this subject. The protocols used differ from study to study, making them not comparable and studies do not tend to reach consensual outcomes. A systematic review investigating the biological effects of 3D-printed resins used in orthodontics concludes that 3D-printed aligners exhibit higher levels of cytotoxicity and genotoxicity than thermoplastic resins, especially if they have not been subjected to a final surface treatment. This prompts the clinician to act with caution, especially when treating young children and growing patients in order to protect them from fertility issues [81].

Another conclusion that we can reach from the studies included in our research for the 3D-printed clear retainers was that the anterior regions of the 3D-printed clear retainers are the most problematic, regardless of print angulation [22]. Williams et al. in particular, concluded that the smooth facial surfaces of central incisors provided greater differences up to 0.480 mm [34].

Deviations of fitting of a 3D-printed clear retainer were attributed to various causes. Cole et al. attributed the greatest deviations of the 3D-printed retainer group to the PMMA resin used, which is very rigid, and assumed that this kind of resin is not the material of choice for this purpose [21]. Naeem also concluded that the print orientation, location of the model on the baseplate, post-processing procedures of the retainers, overexposures of some layers as the build develops because of a clear resin and errors from the CBCT scan affected the accuracy of the aligners [22].

Cole et al. set the accuracy threshold at 0.50 mm somewhat arbitrarily, as it is not clarified by the literature. He relied on studies that have indicated that measurements up to 0.50 mm are clinically agreeable for the appraisal of digital articulation. Naeem set 0.25 mm of maximum deviation as the accuracy threshold according to a study by Johal et al. [82], who tested the fit of thermoformed retainers in vitro and concluded that this threshold was the maximum millimeter deviation. The same clinically acceptable maximum distance was adopted by Williams et al., who relied on a study of Boyd and Vlaskalic [83]. Boyd et al. concluded that a distance of 0.15–0.25 mm is necessary between the aligner and its model cast in order to deliver its forces appropriately. Consequently, Williams et al. considered that deviations up to 0.25 mm were considered clinically acceptable for a retention device. Therefore, an acceptable accuracy threshold is also something that needs to be further examined to be used universally in future studies.

The application of direct 3D-printed retainers in 3D printing pharmaceuticals is another revolutionary technology. Three-dimensional printing has given the capability of manufacturing drug delivery devices such as occlusal splints or retainers with customized design and drug delivery rates. Reports have been made of customized 3D-printed mouthguards loaded with vanillic acid in humans [84]. Another study delivered personalized orthodontic retainers loaded with clonidine hydrochloride for local sustained release of the drug with satisfying results [84,85]. This promising technology could have applications for the treatment of oral diseases as well, such as periodontitis, oral candidiasis, herpetic gingivostomatitis or oral carcinoma. Personalized 3D-printed drug-loaded orthodontic retainers seem to be an emerging technology for the sustained release of drugs [85].

Moreover, 3D-printed fixed retainers seem very promising. The resin-based type of retention is an alternative for patients with metal allergies and aesthetic problems and the material used does not interfere with magnetic resonance imaging (MRI). However, even the metal-type 3D-printed retainers are better than the conventional fixed retainers [86]. The amount of adhesive used can be eliminated because of the shape and size individuality of these devices [24]. Moreover, they have smooth surfaces that accumulate less plaque [35] and their fabrication is time and cost effective [24].

***Strengths and limitations***

This review was constructed on well-established guidelines as outlined in the Materials section. The searching procedure was extensive up to December 2022 and was detailed including every potentially eligible report.

The limitations of the present review might be associated with the nature of the included research and the data characteristics (four in vitro studies and only one prospective clinical trial with a short follow-up of six months). Additionally, the English language restriction can be considered as a weakness. The heterogeneity of protocols among the presented studies and the high risk of bias in the majority of them discouraged the performance of further meta-analysis.

## 5. Conclusions

The transition from conventional methods to a fully digital workflow for the production of 3D-printed retainers is the future trend. Affordable intraoral scanners, 3D printers and free available software make 3D-printing technology more time and cost effective than the conventional techniques. It is also an easier procedure for the practitioner and a more convenient experience, especially for uncooperative or younger patients. However, more in vitro and in vivo clinical trials should be conducted in a well-organized manner in order to clarify some «grey» points concerning the most suitable materials in 3D printing technology: the print angulation and the post-processing procedures, especially for direct-printed clear retainers, as well as the longevity of these appliances in intraoral conditions. The safety of these products should be verified because the majority of the patients involved in orthodontic treatment are growing patients who will keep these retention devices intraorally for the rest of their lives.

## Figures and Tables

**Table 1:** Electronic databases searched, the search terms used and the results for each database.

Database Searched	Search Terms	Results
Medline (via Pubmed)	3D-printed OR three-dimensional-printed orthodontic retainers OR direct-printed retainers.	13
Scopus	3D-printed orthodontic retainers.	126
Cochrane Library	3D-printed orthodontic retainers.	3
Google scholar	3D-printed orthodontic retainers.	2490

**Table 2:** Risk of bias assessment for in vitro studies using the Quin tool. 1: Clearly stated aims/objects; 2: Detailed explanation of sample size calculation; 3: Detailed explanation of sampling technique; 4: Details of comparison group; 5: Detailed explanation of the methodology; 6: Operator details; 7: Randomization; 8: Method of measurement outcome; 9: Outcome assessor Details; 10: Blinding; 11: Statistical analysis; and 12: Presentation of results; H: High; L: Low; U: Unclear.

	Signaling Questions												
Study	1	2	3	4	5	6	7	8	9	10	11	12	Summary
Cole et al., 2019 [38]	L	H	H	H	L	L	U	L	L	H	L	L	H
Naeem et al., 2022 [39]	L	L	H	H	L	H	L	U	L	H	L	L	H
Williams et al., 2022 [40]	L	L	L	H	L	H	L	U	L	H	L	U	H
Firlej et al., 2022 [24]	L	H	H	H	H	H	H	U	U	H	H	H	H

**Table 3:** Risk of bias assessment for the in vivo study using the RoB-2.0 tool.

Study	Bias Arising from the Randomization Process.	Bias Due to Deviations from the Intended Interventions.	Bias Due to Missing Data.	Bias in Measurement of the Outcome.	Bias in Selection of the Reported Result.	Overall Risk
Shah et al., 2022 [35]	High.	Some concerns.	High.	Low.	Some concerns.	High.

## Data Availability

Data is contained within the article.
